# ChloroMitoSSRDB 2.00: more genomes, more repeats, unifying SSRs search patterns and on-the-fly repeat detection

**DOI:** 10.1093/database/bav084

**Published:** 2015-09-25

**Authors:** Gaurav Sablok, G. V. Padma Raju, Suresh B. Mudunuri, Ratna Prabha, Dhananjaya P. Singh, Vesselin Baev, Galina Yahubyan, Peter J. Ralph, Nicola La Porta

**Affiliations:** ^1^Plant Functional Biology and Climate Change Cluster (C3), University of Technology Sydney, PO Box 123, Broadway, NSW 2007, Australia,; ^2^Environmental Biotechnology Platform, Research and Innovation Center, Fondazione Edmund Mach (FEM), IASMA Via Mach 1., 38010 San Michele all'Adige (TN), Italy,; ^3^Department of Computer Science and Engineering, S.R.K.R Engineering College, Chinna Amiram, Bhimavaram 534204, Andhra Pradesh, India,; ^4^Technology Centre, S.R.K.R. Engineering College, Chinna Amiram, Bhimavaram 534204, Andhra Pradesh, India,; ^5^National Bureau of Agriculturally Important Microorganisms (NBAIM) (Indian Council of Agricultural Research), Maunath Bhanjan 275101, Uttar Pradesh, India and; ^6^Department of Plant Physiology and Molecular Biology, University of Plovdiv, 24 Tsar Assen St, 4000 Plovdiv, Bulgaria.

## Abstract

Organelle genomes evolve rapidly as compared with nuclear genomes and have been widely used for developing microsatellites or simple sequence repeats (SSRs) markers for delineating phylogenomics. In our previous reports, we have established the largest repository of organelle SSRs, ChloroMitoSSRDB, which provides access to 2161 organelle genomes (1982 mitochondrial and 179 chloroplast genomes) with a total of 5838 perfect chloroplast SSRs, 37 297 imperfect chloroplast SSRs, 5898 perfect mitochondrial SSRs and 50 355 imperfect mitochondrial SSRs across organelle genomes. In the present research, we have updated ChloroMitoSSRDB by systematically analyzing and adding additional 191 chloroplast and 2102 mitochondrial genomes. With the recent update, ChloroMitoSSRDB 2.00 provides access to a total of 4454 organelle genomes displaying a total of 40 653 IMEx Perfect SSRs (11 802 Chloroplast Perfect SSRs and 28 851 Mitochondria Perfect SSRs), 275 981 IMEx Imperfect SSRs (78 972 Chloroplast Imperfect SSRs and 197 009 Mitochondria Imperfect SSRs), 35 250 MISA (MIcroSAtellite identification tool) Perfect SSRs and 3211 MISA Compound SSRs and associated information such as location of the repeats (coding and non-coding), size of repeat, motif and length polymorphism, and primer pairs. Additionally, we have integrated and made available several *in silico* SSRs mining tools through a unified web-portal for *in silico* repeat mining for assembled organelle genomes and from next generation sequencing reads. ChloroMitoSSRDB 2.00 allows the end user to perform multiple SSRs searches and easy browsing through the SSRs using two repeat algorithms and provide primer pair information for identified SSRs for evolutionary genomics.

**Database URL:** http://www.mcr.org.in/chloromitossrdb

## Introduction

Nuclear and organelle (chloroplast and mitochondrial) genome-based microsatellites or simple sequence repeats (SSRs) markers have been widely used to demonstrate the underpinning differences in genetic patterns and for assessing the phylogenomic and genetic associations between species of particular clade (1–3). In comparison to nuclear genome, organelle genomes have been preferred as a choice for developing such markers taking into account their key features such as conserved pattern of gene order, lack of heteroplasmy, low recombination and substitution rates (4, 5) and relatively small genome size, thus making organelle genomes as a model system for developing rapid source of markers for understanding the phylogenomics and species delineation (6, 7). Recent advances in the sequencing technologies and availability of the low-cost sequencing strategies, isolation and subsequent sequencing of high coverage organelle genomes for the understanding of phyletic patterns of sequence variation, and developing of species-specific and conservation markers, have been revolutionized (6, 7).

Development of species-specific or cross-species transferrable amplifiable SSRs markers from organelle genomes has been shown as a discovery to application approach realizing the ease of development, high rate of transferability and variation (8, 9). Realizing the ease of application, organelle genome markers have been widely applied for resolving the patterns of molecular evolutions, demographic and phylo-geographical diversity and to understand the genetic basis of species adaptions ranging from *Pinus* (forest species) (8) to *Oryza sativa* (Monocots). (10, 11) Recent reports demonstrate the *in* *silico* identification of SSRs in organelle genomes of various organisms including plants. (12–15) However, the reported studies have focused on either relatively small number of organelle genomes or organelle genome representing a specific clade. Previously developed organelle repositories such as FUGOID (16), primer respository for chloroplast genomes (17), GOBASE (18) and AT_CHLORO (19), although provides a wide array of curated information on certain aspects of organelle genomics, they lack SSRs information, which motivated the earlier establishment of ChloroMitoSSRDB (9), as an integrated open-access portal for browsing SSRs patterns from organelle genomes across several clades of organism. Following this approach, two SSRs repositories, namely MitoSatPlant (15) and ChloroSSRdb (20) have been developed specifically focusing on plant species. However, despite the development of these plant centric repositories, a unifying portal for the comparative visualization of repeats incorporating several organisms and ‘on-the-fly’ repeat mining from either the gene or genome-based organelle assemblies or the next generation sequencing (NGS) reads is still lacking. The mere lack of this knowledge gap motivated us to develop ChloroMitoSSRDB 2.00, a sequel update to the ChloroMitoSSRDB.

In the present research, we present ChloroMitoSSRDB 2.00, an update to the previously established microsatellite (SSRs) repository ChloroMitoSSRDB, by systematically analyzing and adding SSRs entries for additional 191 chloroplast and 2102 mitochondrial genomes thus displaying a total of 4454 organelle genomes. Additionally, ChloroMitoSSRDB 2.00 provides a web-accessible unified portal for the identification of the maximal/perfect/imperfect SSRs repeats using IMEx (Imperfect Microsatellite Extractor) (21), MISA (MIcroSAtellite identification tool) and REPuter (22) either from the sequenced organelle genomes or directly from NGS reads, using the PAL finder. (23, 24) The present version of ChloroMitoSSRDB 2.00 contains 4454 organelle genomes which includes 370 chloroplast genomes, and 4084 mitochondrial genomes displaying a total of 40 653 IMEx Perfect SSRs (11 802 Chloroplast Perfect SSRs and 28 851 Mitochondria Perfect SSRs), 275 981 IMEx Imperfect SSRs (78 972 Chloroplast Imperfect SSRs and 197 009 Mitochondria Imperfect SSRs), 35 250 MISA Perfect SSRs and 3211 MISA compound SSRs.

Availability of the SSR mining tools under a common portal plus a systematically curated in-house database, we believe that ChloroMitoSSRDB 2.00 will serve as a portal for the identification and assessment of organelle repeat evolution, developing species-specific markers, identifying estimates of genetic diversity based on organelle marker abundances, phylogenomics and other organelle-based genotyping approaches. To the best of our knowledge, this is the first integrated portal, which catalogs the repeat search pattern for thousands of organelle genomes, across diverse phylogenetic clades in a systematic manner along with the ‘on-the-fly’ availability of the organelle repeat search tools for organelle genomics, which is accessible via web-interface.

## Materials and methods

### Update to the genome data retrieval and pattern search

To update the existing ChloroMitoSSRDB database, additional 2293 organelle representing 2102 mitochondrial and 191 chloroplast genome files (GBK, FNA, FAA, GFF and PTT) were systematically downloaded from NCBI RefSeq database release 63 (www.ncbi.nlm.nih.gov/). Additionally, each organelle genome was scanned for SSRs patterns using two different tools: IMEx (21) and MISA (available from http://pgrc.ipk-gatersleben.de/misa/misa.html). IMEx (21) algorithm allows searching of the SSRs using a sliding window algorithm to identify regions with a repetitive stretch of a particular nucleotide motif, either stretched perfectly or with some level of imperfection. SSRs mined using the IMEx algorithm were further linked to the respective coding or non-coding regions on the basis of the genic information available from the respective GenBank files (GBK and PTT). For IMEx pattern search, we used the previously applied length threshold parameters (Mono-, 12; Di-, 6; Tri-, 4; and for Tetra- to Hexa- repeats, a minimum stretch of three minimum repetitions) (9). For identifying the imperfect repeats, the imperfection percentage, which indicates the level of imperfection (p%), is set to 10%.

Additionally, each organelle genome has been simultaneously analyzed using MISA algorithm tool (MISA; http://pgrc.ipk-gatersleben.de/misa/misa.html), which allows the detection of the perfect, imperfect and compound repeats. For a stretch of nucleotide to be classified as the SSRs using MISA, a minimum length of ≥12 bp for Mono-, ≥6 bp for Di-, ≥4 bp for Tri- and ≥3 bp for Tetra-, Penta- and Hexa-nucleotide repeats were used as length thresholds, respectively. For the identification of the compound SSRs, we kept the minimum distance between any two identified SSRs as 100 bp. Following the identification of the SSRs using MISA, primer pairs for the each set of the MISA identified SSRs, primer pairs were designed using Primer3 with settings PRIMER_PRODUCT_SIZE_RANGE=100-280 and PRIMER_MAX_END_STABILITY=250. In order to make SSRs markers readily available for downstream analysis, we integrated only those MISA SSRs markers, which have the corresponding primer pair information. The genome composition and the repeat occurrence graphs were generated dynamically using HighCharts, a JavaScript chart-drawing library (www.highcharts.com/products/highcharts). ChloroMitoSSRDB 2.00 is hosted on a 64-bit Linux server pre-installed with Apache (http://www.apache.org/) and PHP (http://www.php.net/).

### Web-based repeat detection

As compared with ChloroMitoSSRDB, ChloroMitoSSRDB 2.00 provides additional web-based SSRs identification using IMEx, MISA and REputer (22), which have been widely used for SSRs identification from organelle genomes. The web-based SSR identification has been customized as per the parameters widely described for organelle genomes. For example, IMEx (21) will be executed with setting Mono-, 12; Di-, 6; Tri-, 4; Tetra to Hexa-3 and p%-10% for identifying imperfect repeats using NC_007194.fna 1 1 1 2 2 3 10 10 10 10 10 10 12 6 4 3 3 3 100 1 1 1 10 3 0 NC_007194.ptt. MISA can be used with or without primer designing using the length threshold and primer3 settings, as described in the Materials and methods section. For the identification of compound SSRs, default value of 100 has been kept as the minimum distance between two SSRs stretches in the web-based SSR portal. Additionally, REPuter (22) can be used with settings specific to organelle genomes (-f = compute maximal forward repeats, -p = compute maximal palindromes, -r = compute maximal reverse repeats, -c = compute maximal complemented repeats, -l 30 = specify that repeats must have the given length, -h 3 = search for repeats up to the given hamming distance, -s = show the string content of the maximal repeats). In addition to the already identified SSRs from the assembled organelle genomes available so far, ChloroMitoSSRDB 2.00 provides ‘on-the-fly’ identification of the Potentially Amplifiable Loci (PALs) from Illumina sequencing reads using PAL finder available from http://sourceforge.net/projects/palfinder/ and as previously described (23, 24).

## Results and discussions

### Updated integrated structure and functionalities of ChloroMitoSSRDB 2.00

**Figure 1.**
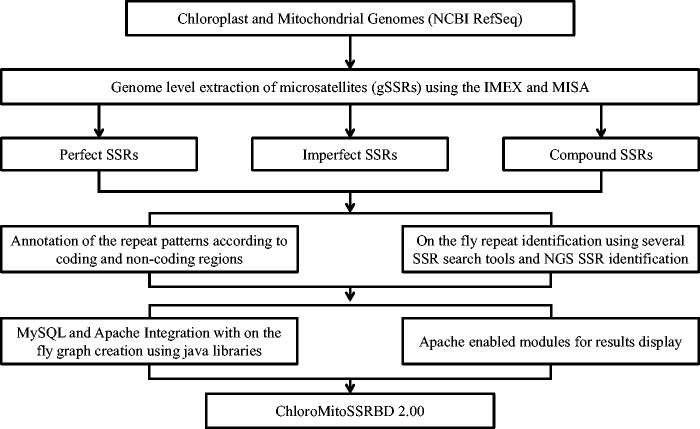
Updated enhanced illustrated view of the flow of the information of the data in ChloroMitoSSRDB 2.00.

**Figure 2.**
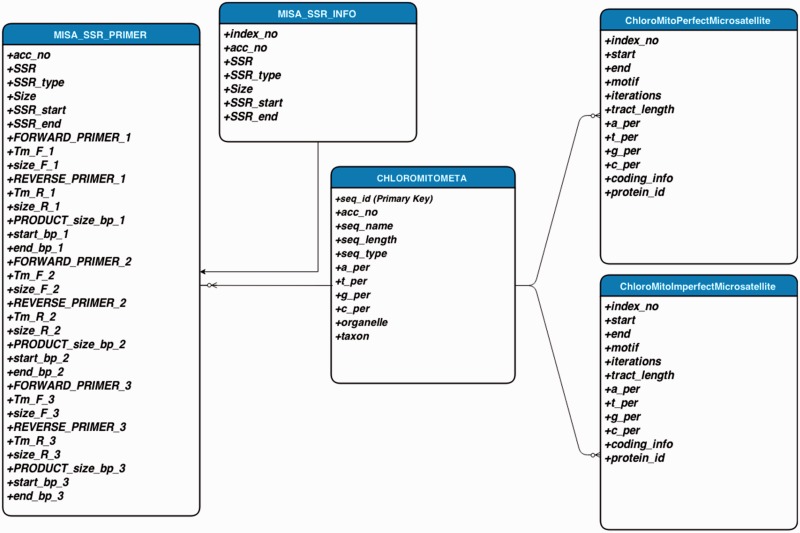
Entity-relationship model diagram showing the layout of the database schema in ChloroMitoSSRDB 2.00.

A sketch of the updated computational workflow of ChloroMitoSSRDB 2.00 is given in Figure 1. The comprehensive workflow of the ChloroMitoSSRDB has been updated while using the same relational database management system, MySQL (http://www.mysql.com/). In this update, identified SSRs from the IMEx and MISA algorithms for each of the organelle genomes were integrated for visualization through respective algorithm specific pages. The relational database system of ChloroMitoSSRDB 2.00 has been updated by adding primer-pair information corresponding to MISA identified SSRs, as a separate query field. Table 1 describes the meta-data information embedded in the ChloroMitoSSRDB 2.00. Query fields for IMEx have been updated to integrate the MISA identified SSRs and are displayed in Table 2. To make the search pattern unified across all genomes, MISA-identified SSRs have been linked to each genome using the query fields given in Table 3. Visualization of the entity-relationship model between the hierarchical query classifications is presented in Figure 2 and as Supplementary Figures S1 and S2.

**Table 1.** Structure of table ‘chloromitometa’ that stores the meta-information of all the mitochondrial and chloroplast genomes

Information	Field	Data type	Key	Example
Accession number	acc_no	int(11)		5881414, 110189662
Sequence ID	seq_id	varchar(11)	PRI	NC_000834, AC_000022
Sequence name	seq_name	varchar(500)		*Rattus norvegicus* strain Wistar mitochondrion, *Porphyra purpurea* chloroplast
Sequence type	seq_type	varchar(50)		Complete genome, complete sequence
Sequence length	seq_length	int(11)		16 613 bp, 7686 bp
Nucleotide composition of A	a_per	Float		33.06%
Nucleotide composition of T	t_per	Float		41.87%
Nucleotide composition of G	g_per	Float		13.58%
Nucleotide composition of C	c_per	Float		11.49%
Organelle type	Organelle	char(1)		M (for Mitochondrion), C (Chloroplast)
Taxon ID	Taxon	int(11)		85636, 6334

**Table 2.** Structure of the tables ‘chloromitoperfectmicrosatellite’ and ‘chloromitoimperfectmicrosatellite’ that stores the repeat information detected by IMEx of all perfect and imperfect microsatellites of mitochondrial and chloroplast genomes

Information	Field	Data type	Key	Example
Sequence ID	index_no	varchar(11)	PRI	NC_000834, AC_000022
Starting co-ordinate of SSR	Start	int(11)	PRI	172, 12843
Ending co-ordinate of SSR	End	int(11)	PRI	182, 12885
Motif (repeating unit)	Motif	varchar(10)		AT, G, CAAC
Number of repetitions	Iterations	int(5)		3, 7
Length of repeat tract	tract_length	int(11)		12 bp, 18 bp
Nucleotide composition of A	a_per	Float		50.00%
Nucleotide composition of T	t_per	Float		0.00%
Nucleotide composition of G	g_per	Float		33.33%
Nucleotide composition of C	c_per	Float		16.67%
Repeat position Info	coding_info	varchar(50)		Coding (if repeat in coding region) or NULL (if outside)
Protein ID (if repeat in coding region)	protein_id	int(11)		110189664 (if repeat in coding region) or 0 (if non-coding)
Imperfection % of the tract	Imperfection	Float		9%, 0%
Alignment Line 1	Alignment_line1	Text		TTAA-TAATTAA
Alignment Line 2	Alignment_line2	Text		**** *******
Alignment Line 3	Alignment_line3	Text		TTAATTAATTAA

The last four columns (imperfection, alignment_line1, alignment_line2 and alignment_line3) are present only in the table storing imperfect microsatellites (chloromitoimperfectmicrosatellite).

**Table 3.** Structure of the table ‘misa_ssr_info’ that stores the repeat information detected by MISA of all perfect and compound microsatellites of mitochondrial and chloroplast genomes

Information	Field	Data type	Key	Example
Accession number	acc_no	int(11)		5881414, 110189662
Sequence ID	index_no	varchar(11)	PRI	NC_000834, AC_000022
Motif with iteration count	SSR	varchar(255)		(AT)4
Type of repeat	SSR_type	varchar(5)		p1, (mono), p2 (di), p3 (tri) etc, c and c* (compound)
Size	int(4)	int(4)		31, 20
Starting co-ordinate of SSR	SSR_start	int(7)	PRI	172, 12843
Ending co-ordinate of SSR	SSR_end	int(7)	PRI	182, 12885

**Figure 3.**
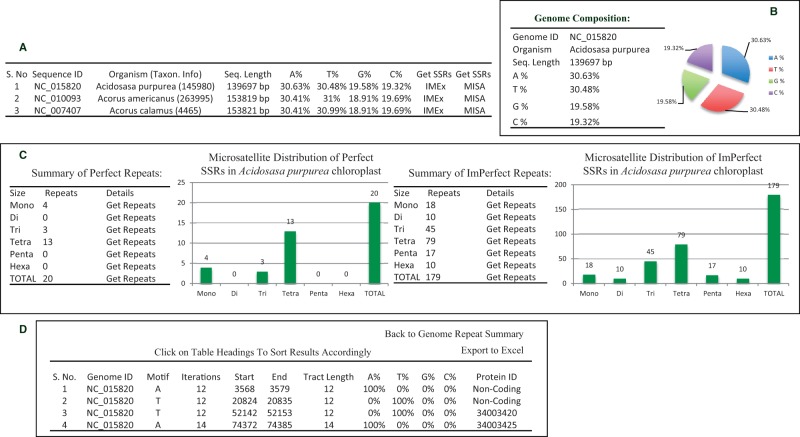
Webpage of ChloroMitoSSRDB 2.00 describing repeat summary of *Acidosasa purpurea* chloroplast extracted from IMEx. (**A**) Details of chloroplast microsatellites. (**B**) Repeat summary of *Acidosasa purpurea* chloroplast repeat extracted by IMEx and nucleotide composition of *Acidosasa purpurea* chloroplast. (**C**) Summary of perfect and imperfect repeats in *Acidosasa purpurea* chloroplast along with graphical distribution. (**D**) Mono-nucleotide perfect repeats of *Acidosasa purpurea* chloroplast where coding repeats in Protein ID column are linked to NCBI.

In this update, ChloroMitoSSRDB 2.00 provides the tabular view of the analyzed chloroplast and mitochondrial genomes, which are alphabetically sorted, and can be browsed according to choice of selected organelle (chloroplast, (http://www.mcr.org.in/chloromitossrdb/chloro_browse.php; mitochondrial, http://www.mcr.org.in/chloromitossrdb/mito_browse.php) genomes. Each organelle genome has been hyperlinked to the corresponding taxonomy record as previously available in ChloroMitoSSRDB. Organelle (chloroplast and mitochondrial) genome-specific pages offer options for the end users to systematically browse through the results of the IMEx (21) and MISA repeat mining algorithms such as chloroplast (http://www.mcr.org.in/chloromitossrdb/chloro_browse.php) and mitochondrial (http://www.mcr.org.in/chloromitossrdb/mito_browse.php) genomes (Figure 3).

**Figure 4.**
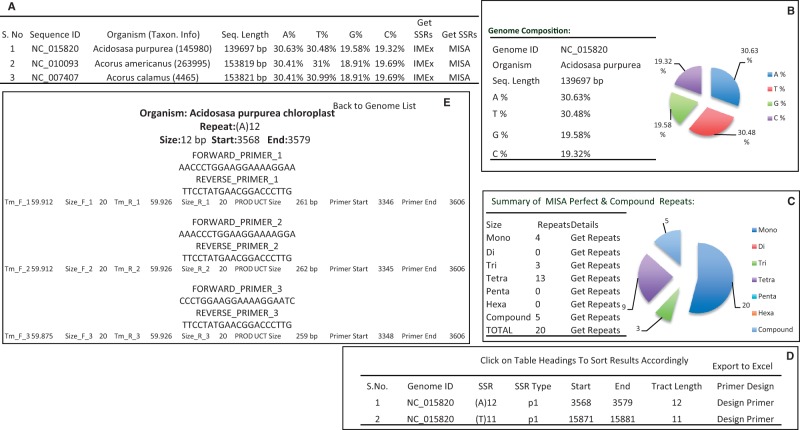
Repeat summary of *Acidosasa purpurea* chloroplast repeat extracted by MISA. (**A**) Details of chloroplast microsatellites. (**B**) Repeat summary of *Acidosasa purpurea* chloroplast repeat extracted by MISA and nucleotide composition of *Acidosasa purpurea* chloroplast. (**C**) Summary of MISA perfect and compound SSRs in *Acidosasa purpurea* chloroplast in tabular and graphical manner. (**D**) Detail information about perfect and compound SSRs in *Acidosasa purpurea* chloroplast. (**E**) Primers list and associated information available for any particular SSR.

ChloroMitoSSRDB 2.00 is PHP enabled and connects with the backhand MySQL server allowing for the rapid visualization of the SSRs across several organelle genomes. It has been configured to meet the need of end users, working toward the SSR characterization and developing genus species markers. To enable rapid searches, enhanced PHP-based web functionalities have been added to allow browsing simultaneously the results from IMEx (Figure 3A–D), or MISA (Figure 4A–E). A complete schema of the browsing functionalities is given in Figures 3A–D and 4A–E. Organelle-specific (chloroplast or mitochondrial) genome web pages show the integrated curated information such as distribution of the repeat types (Figures 3A and C and 4A and C), length of the motifs and their positions (coding or non-coding repeats as derived from the PTT files) (Figure 3D), nucleotide composition (Figures 3B and 4B), as derived from IMEx and MISA in two separate web-interface functionalities, displaying the information on the repeat statistics (Figures 3 and 4).

Clickable IMEx and MISA links display the associated repeat information in the form of pie charts and repeat tables (Figures 3C and 4C). For example, SSRs information display page, showing results of IMEx algorithm (IMEx: http://www.mcr.org.in/chloromitossrdb/genome_repeat_summary.php?id=NC_015820 and SSRs information display page showing repeat information and primer pairs from MISA: http://www.mcr.org.in/chloromitossrdb/misa_genome_repeat_summary.php?id=NC_021932). In line with the previous version of the database, each organelle genome page displays the genome composition (A-, T-, G-, C- counts, etc.) (Figures 3B and 4B) and the systematic sorted information on sequence ID start and end coordinates of the repeats, the repeating motif, number of iterations, total tract length, nucleotide composition of the SSRs and linking of the repeat information to the coding and non-coding capacity and availability of the corresponding primer pair, in case of IMEX and MISA SSRs (Figures 3D and 4D; http://www.mcr.org.in/chloromitossrdb/get_repeats.php?id=NC_015820&size=1&type=0&org=0). Additionally, in case of repeats localized with in the genic regions (Figure 3D), the coding repeats have been hyperlinked to the NCBI gene records.

As the ancillary focus of this update to the database, is to provide ‘primer pairs’ information for the repeats identified using MISA algorithm. Keeping in view the goal of effective integration of the MISA repeats, repeats identified using the MISA algorithm were sorted based on motif for each genome (Figure 4D; http://www.mcr.org.in/chloromitossrdb/misa_get_repeats.php?id=NC_009268&size=1&type=0&org=0), and each identified SSRs motif has been hyperlinked to the corresponding primer pair information (Figure 4E; http://www.mcr.org.in/chloromitossrdb/primers.php?id=NC_009268&start=4251&end=4268) (Table 4; Figure 4E). Availability of the primer pair information, associated with each repeats is critical to this, update as availability of the primer pair information will help end-user to develop ‘ready to go’ primers that can be used for diversity estimates. ChloroMitoSSRDB 2.00 also provides users with an option to export the search results obtained from two different repeat mining algorithms, IMEx (21) and MISA, as well as the repeat information in EXCEL compatible format, to utilize the information for further downstream processing of the observed repeats in user-specified organelle genome. Additionally, availability of the IMEx (21) SSRs alignments and their consensus allows users to query and identify biased patterns of evolution of certain repeats across evolutionary clades of organisms to understand the phyletic pattern of SSRs evolution.

**Table 4.** Structure of the table ‘misa_ssr_primer’ that stores the primer information of microsatellites of mitochondrial and chloroplast genomes detected by MISA

Information	Field	Data Type	Key	Example
Accession number	acc_no	int(11)	PRI	5881414, 110189662
Motif with iteration count	SSR	varchar(255)		(AT)4
Type of repeat	SSR_type	varchar(5)		p1, (mono), p2 (di), p3 (tri) etc, c and c* (compound)
Size	int(4)	int(4)		31, 20
Starting co-ordinate of SSR	SSR_start	int(7)	PRI	172, 12843
Ending co-ordinate of SSR	SSR_end	int(7)	PRI	182, 12885
Forward primer 1	FORWARD_PRIMER_1	varchar(30)		AAAAAGGCCCCTTCCCCC
Melting temperature for forward primer 1	Tm_F_1	varchar(6)		59.463
Size of forward primer 1	size_F_1	int(6)		18
Reverse primer 1	REVERSE_PRIMER_1	varchar(30)		GCGCCTAAGGATCCTGTGAG
Melting temperature for reverse primer 1	Tm_R_1	varchar(6)		60.25
Size of reverse primer 1	size_R_1	int(6)		20
Product size (in bp)	PRODUCT_size_bp_1			220
Starting co-ordinate of primer 1	start_bp_1			6256
Ending co-ordinate of primer 1	end_bp_1			6475

The last nine columns of the table will be repeated for reverse primer 1, forward primer 2, reverse primer 2, forward primer 3 and backward primer 3.

### Web-based on-the-fly repeat detection in ChloroMitoSSRDB 2.00

**Figure 5.**
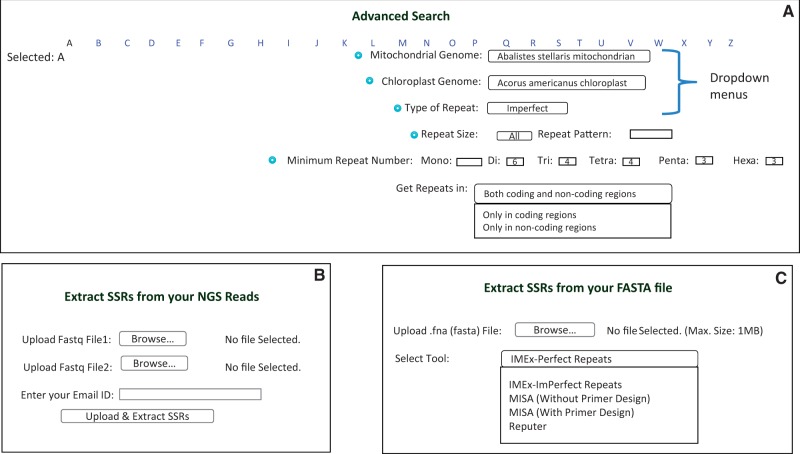
Advanced search and SSR extraction options in ChloroMitoSSRDB. (**A**) Advanced search page. (**B**) Page providing facility to extract SSRs from NGS Reads. (**C**) Page providing option of SSRs extraction in user-provided FASTA sequence.

In addition to the previous ChloroMitoSSRDB functionalities, such as search patterns according to organelle, type of repeat pattern (perfect or imperfect) and size and length of repeat motif, we present a new advanced search panel with an enhanced drop-down box which is now available showing several additional search patterns based on coding and non-coding classification patterns (Figure 5A). With the rapid development of NGS technologies, significant advances in sequencing and assembling the chloroplast regions and evolution of the repeat content have been investigated (25, 26). NGS provides a cost-efficient way of genomic representation and developing SSRs markers for model and non-model species. Recently, identifying markers from the sequencing reads has gained widespread interest as genetic markers, and has also been shown to be resource-intensive markers for species discrimination (6, 7). Several pipelines such as PAL finder (23, 24), High SSR (27), iMSAT (28) and SSR_pipeline (29) have been recently developed to identify amplifiable polymorphic markers from the NGS reads, thus mitigating the necessity of the transcriptome/genome assembly. In addition to the mining of the repeats from the assembled genome, ChloroMitoSSRDB 2.00 also provides web-based extraction of the PAL under ‘Extract SSRs from NGS reads’: http://www.mcr.org.in/chloromitossrdb/ngs_upload.php using PAL finder (23, 24) from to identify repeats from chloroplast or mitochondrial NGS reads along with the corresponding primer pair information (Figure 5B). To facilitate the SSRs extraction from the assembled genome, ChloroMitoSSRDB 2.00 provides ‘on-the-fly’ extraction utility ‘Extract SSRs’ (http://www.mcr.org.in/chloromitossrdb/extract.php), where user can analyze the SSRs from their assembled genome using any of the repeat mining algorithm such as IMEx (21), REputer (22) or MISA with or without primer design (Figure 5C).

## Conclusion

In conclusion, ChloroMitoSSRDB 2.00 provides an enhanced visualization and unified update to the previously developed integrated repository of the organelle genome by integrating SSRs patterns from two different tools IMEx and MISA. ChloroMitoSSRDB 2.00 also overcomes the limitations of the SSR search pattern for the user-defined gene-based or the whole-genome-based sequences by integrating three widely implemented tools for SSRs search pattern. In future work, we plan to establish a sequence-based retrieval for the complete NGS-based SSRs patterns for marker development across a wide range of organelle genomes. We believe that the enhanced version of the database portal along with the comparative integration of two repeats mining algorithms, and ‘on-the-fly’ repeat extraction will support a wide range of the organelle genomics community and will serve as a platform for wider organelle genome-wide SSR explorations.

## Author Contributions

GS designed the study and carried out the analysis; GVPR, SBM updated the database in discussion with GS; RP and DPS helped in the database update; GS wrote the manuscript; VB, GY, PJR and NLP provided revisions to the manuscript.

## Supplementary Data

Supplementary data are available at *Database* Online.

Supplementary Data
