# Supplementary material for: ChloroMitoSSRDB 2.00: more genomes, more repeats, unifying SSRs search patterns and on-the-fly repeat detection
Source: Database (Oxford). 2015 Sep 25;2015:bav084. doi: 10.1093/database/bav084 (PMC4584093; doi:10.1093/database/bav084)
Supplement: Supplementary Data [file supp_2015_bav084_index.html]

ChloroMitoSSRDB 2.00: more genomes, more repeats, unifying SSRs search patterns and on-the-fly repeat detection — Supplementary Data 

# ChloroMitoSSRDB 2.00: more genomes, more repeats, unifying SSRs search patterns and on-the-fly repeat detection

## Supplementary Data

files

- Supplementary Data - zip file
