# Supplementary figures and images for: ChloroMitoSSRDB 2.00: more genomes, more repeats, unifying SSRs search patterns and on-the-fly repeat detection
Source: Database (Oxford). 2015 Sep 25;2015:bav084. doi: 10.1093/database/bav084 (PMC4584093; doi:10.1093/database/bav084)

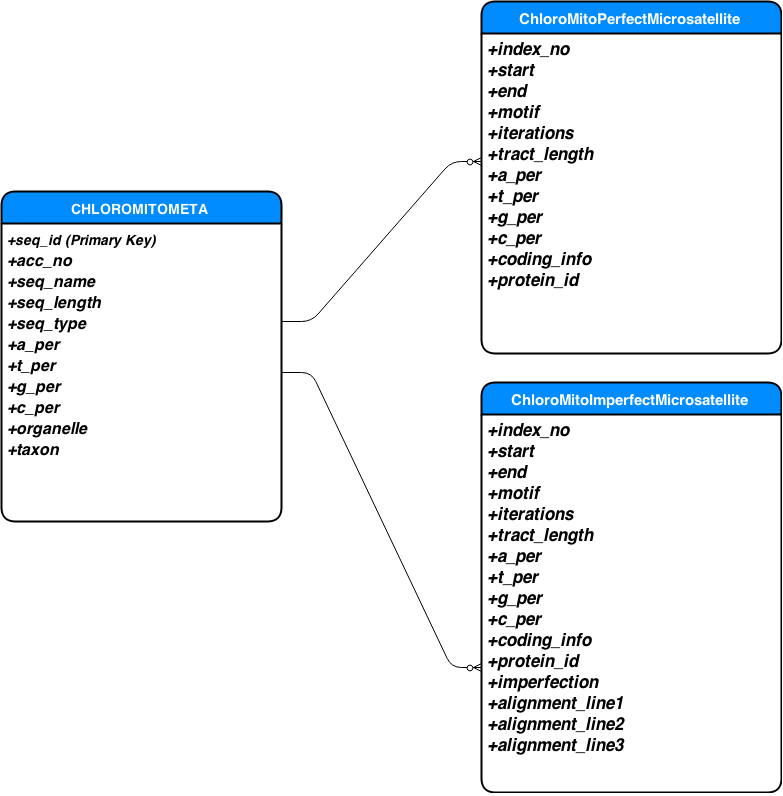

Supplement: Supplementary Data [file supp_bav084_suppl_data.zip › IMEx_ER_Supplementary_File_1.png]

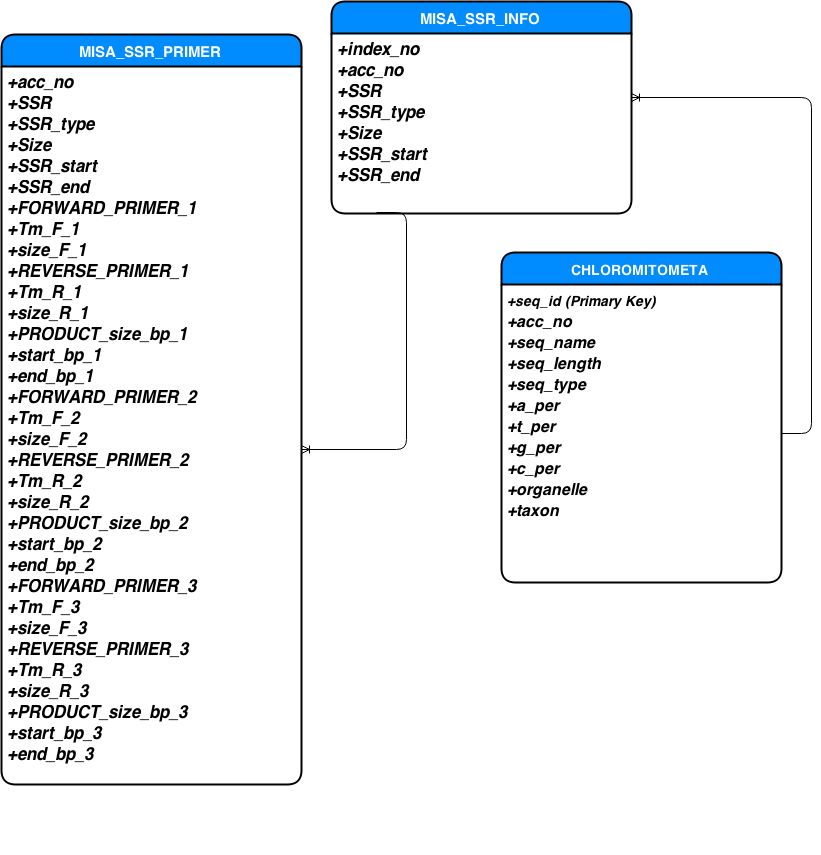

Supplement: Supplementary Data [file supp_bav084_suppl_data.zip › MISA_ER_Supplementary_File_2.png]
