# Supplementary material for: ChloroMitoSSRDB 2.00: more genomes, more repeats, unifying SSRs search patterns and on-the-fly repeat detection
Source: Database (Oxford). 2015 Sep 25;2015:bav084. doi: 10.1093/database/bav084 (PMC4584093; doi:10.1093/database/bav084)
Supplement: Supplementary Data [file supp_bav084_suppl_data.zip › Supplementary_File_1andFile_2.doc]

Supplementary File:

Supplementary Fig.1: ER diagram of IMEx architecture in ChloroMitoSSRDB 2.00

Supplementary Fig.2: ER diagram of MISA architecture in ChloroMitoSSRDB 2.00
